# Transient expression in *Nicotiana benthamiana* for rapid functional analysis of genes involved in non‐photochemical quenching and carotenoid biosynthesis

**DOI:** 10.1111/tpj.13268

**Published:** 2016-09-15

**Authors:** Lauriebeth Leonelli, Erika Erickson, Dagmar Lyska, Krishna K. Niyogi

**Affiliations:** ^1^ Howard Hughes Medical Institute Department of Plant and Microbial Biology University of California Berkeley CA 94720‐3102 USA; ^2^ Molecular Biophysics and Integrated Bioimaging Division Lawrence Berkeley National Laboratory Berkeley CA 94720 USA

**Keywords:** non‐photochemical quenching, PSBS, carotenoid biosynthesis, xanthophyll cycle, transient assay, photosynthesis, *Nicotiana benthamiana*, *Nannochloropsis*, *Thalassiosira*, lutein epoxide

## Abstract

Plants must switch rapidly between light harvesting and photoprotection in response to environmental fluctuations in light intensity. This switch can lead to losses in absorbed energy usage, as photoprotective energy dissipation mechanisms can take minutes to hours to fully relax. One possible way to improve photosynthesis is to engineer these energy dissipation mechanisms (measured as non‐photochemical quenching of chlorophyll *a* fluorescence, NPQ) to induce and relax more quickly, resulting in smaller losses under dynamic light conditions. Previous studies aimed at understanding the enzymes involved in the regulation of NPQ have relied primarily on labor‐intensive and time‐consuming generation of stable transgenic lines and mutant populations – approaches limited to organisms amenable to genetic manipulation and mapping. To enable rapid functional testing of NPQ‐related genes from diverse organisms, we performed *Agrobacterium tumefaciens*‐mediated transient expression assays in *Nicotiana benthamiana* to test if NPQ kinetics could be modified in fully expanded leaves. By expressing *Arabidopsis thaliana* genes known to be involved in NPQ, we confirmed the viability of this method for studying dynamic photosynthetic processes. Subsequently, we used naturally occurring variation in photosystem II subunit S, a modulator of NPQ in plants, to explore how differences in amino acid sequence affect NPQ capacity and kinetics. Finally, we functionally characterized four predicted carotenoid biosynthesis genes from the marine algae *Nannochloropsis oceanica* and *Thalassiosira pseudonana* and examined the effect of their expression on NPQ in *N. benthamiana*. This method offers a powerful alternative to traditional gene characterization methods by providing a fast and easy platform for assessing gene function *in planta*.

## Significance Statement

Transient expression of genes in *Nicotiana benthamiana* has become a method of choice to quickly assess gene function *in planta*. Here we illustrate the potential of transient assays in *N. benthamiana* to provide a medium‐throughput platform for assessing gene function with regards to photosynthetic processes. A promising system is described for studying the regulation of NPQ dynamics toward minimizing energy losses and improving photosynthetic yields.

## Introduction

Conservative projections of population growth have indicated that by 2050 the world's population will exceed 9.2 billion (Raftery *et al*., 2012). This population forecast portends an increase in crop demand for both food and industrial uses–a need which must be met by improving crop yields, as sources of arable land are limited (Wu *et al*., 2014). This foreseeable challenge prompts the question: How can we engineer plants to be more productive?

With respect to theoretical maximum yields of sunlight to biomass conversion, plants use only one‐third of the light energy available for photochemistry (Zhu *et al*., 2010). This inefficient energy usage is the combinatorial result of limited light penetrance into the canopy as well as photoprotective mechanisms that dissipate absorbed energy as heat during periods of light saturation (Melis, 2009; Endo *et al*., 2014). The latter of these two factors is referred to as non‐photochemical quenching of chlorophyll *a* fluorescence (NPQ). NPQ is vital for protecting plants during periods of high light stress, especially in environments with fluctuating light (Külheim *et al*., 2002). However, sustained NPQ can result in wasteful dissipation of light energy in limiting light (Zhu *et al*., 2004; Murchie and Niyogi, 2011), and engineering of NPQ kinetics and capacity present attractive targets for improving photosynthetic yields (Zhu *et al*., 2010; Long *et al*., 2015).

Several different excitation quenching mechanisms contribute to what is collectively known as NPQ. These components include feedback de‐excitation (qE), zeaxanthin‐dependent quenching (qZ), photoinhibition (qI), and state transitions (qT), which are distinguished by the timescales on which they act (Müller *et al*., 2001). The most rapidly responding quenching component, qE, is induced and relaxes over the course of seconds to minutes and requires zeaxanthin formation, a pH gradient and, in plants, the photosystem II subunit S (PSBS) protein (Demmig‐Adams *et al*., 1996; Niyogi *et al*., 1998; Li *et al*., 2000, 2002a). In Arabidopsis, zeaxanthin is a product of the xanthophyll cycle and its accumulation is regulated by the antagonistic activities of two enzymes, violaxanthin de‐epoxidase (AtVDE) and zeaxanthin epoxidase (AtZEP) (Figure 2a). In excess light, a pH gradient builds up across the thylakoid membrane and the resulting low lumen pH activates AtVDE, which catalyzes the conversion of violaxanthin to zeaxanthin via the intermediate antheraxanthin (Yamamoto *et al*., 2004). This acidification of the lumen also activates AtPSBS, which is thought to act as a pH sensor (via protonation of two conserved lumen‐facing glutamate residues) that facilitates the induction of qE (Li *et al*., 2002b, 2004). The exact role of AtPSBS remains unknown, but its presence is required for qE *in vivo*, and its importance is underscored by its existence in all photosynthetic land plants studied to date (Bonente *et al*., 2008).

Mechanistic insights into NPQ have been obtained by studying mutants isolated from *Arabidopsis thaliana* and *Chlamydomonas reinhardtii*, however many other photosynthetic organisms independent of the green lineage perform NPQ (Goss and Lepetit, 2015). These organisms, like the diatom *Thalassiosira pseudonana* and the eustigmatophyte *Nannochloropsis oceanica*, contain a variety of different carotenoids and proteins involved in light harvesting and photoprotection (Brown, 1987; Coesel *et al*., 2008; Vieler *et al*., 2012). This rich biodiversity presents an opportunity to discover new mechanisms of NPQ, along with the genes involved. To this end, advancements in next‐generation sequencing (NGS) have produced a burst of genome sequence information to aid with gene predictions. However, basic biological tools to study gene function in emerging model organisms are still in the developmental phase, stalling efforts to fully characterize these genes.

In an effort to circumvent this bottleneck and characterize potentially interesting genes involved in energy dissipation and carotenoid biosynthesis, we took advantage of a system that has been used extensively by the plant pathology community: transient *Agrobacterium tumefaciens*‐mediated gene expression in *Nicotiana benthamiana* (Rossi *et al*., 1993; Kapila *et al*., 1997; Van der Hoorn *et al*., 2000; Armbruster *et al*., 2016). This system exploits the natural ability of *A. tumefaciens* to genetically modify plant cells, enabling expression of candidate genes *in planta* in a matter of days. This system is particularly well suited for the study of algal genes involved in carotenoid biosynthesis and photosynthesis, as introduced genes can use endogenous pigments as substrates as well as existing photosynthetic machinery as a scaffold. In addition, the activity of genes expressed in the same leaf can be directly compared, because proteins accumulate in the same background.

In this study, we tested the viability of transient expression for modifying dynamic chloroplast processes by examining the effects of *AtVDE*,* AtZEP*, and *AtPSBS* over‐expression on NPQ in fully expanded *N. benthamiana* leaves. We then used this method to directly compare the kinetics and quenching capacity resulting from expression of *PSBS* homologs from an alga, a bryophyte, and a vascular plant (*C. reinhardtii*,* Physcomitrella patens*, and *A. thaliana*, respectively) and showed that PSBS from *P. patens* confers the highest quenching capacity of those tested. Finally, we used the *N. benthamiana* platform to explore the function of four predicted carotenoid biosynthetic genes from emerging model organisms *T. pseudonana* and *N. oceanica*. Several of these proteins demonstrated predicted activity, whereas others, specifically the zeaxanthin epoxidase homolog from *N. oceanica* (*NoZEP1*), showed relaxed substrate specificity and formed lutein epoxide when expressed *in planta*.

## Results and Discussion

### NPQ in *N. benthamiana* is altered by the transient expression of Arabidopsis genes

**Figure 1:**
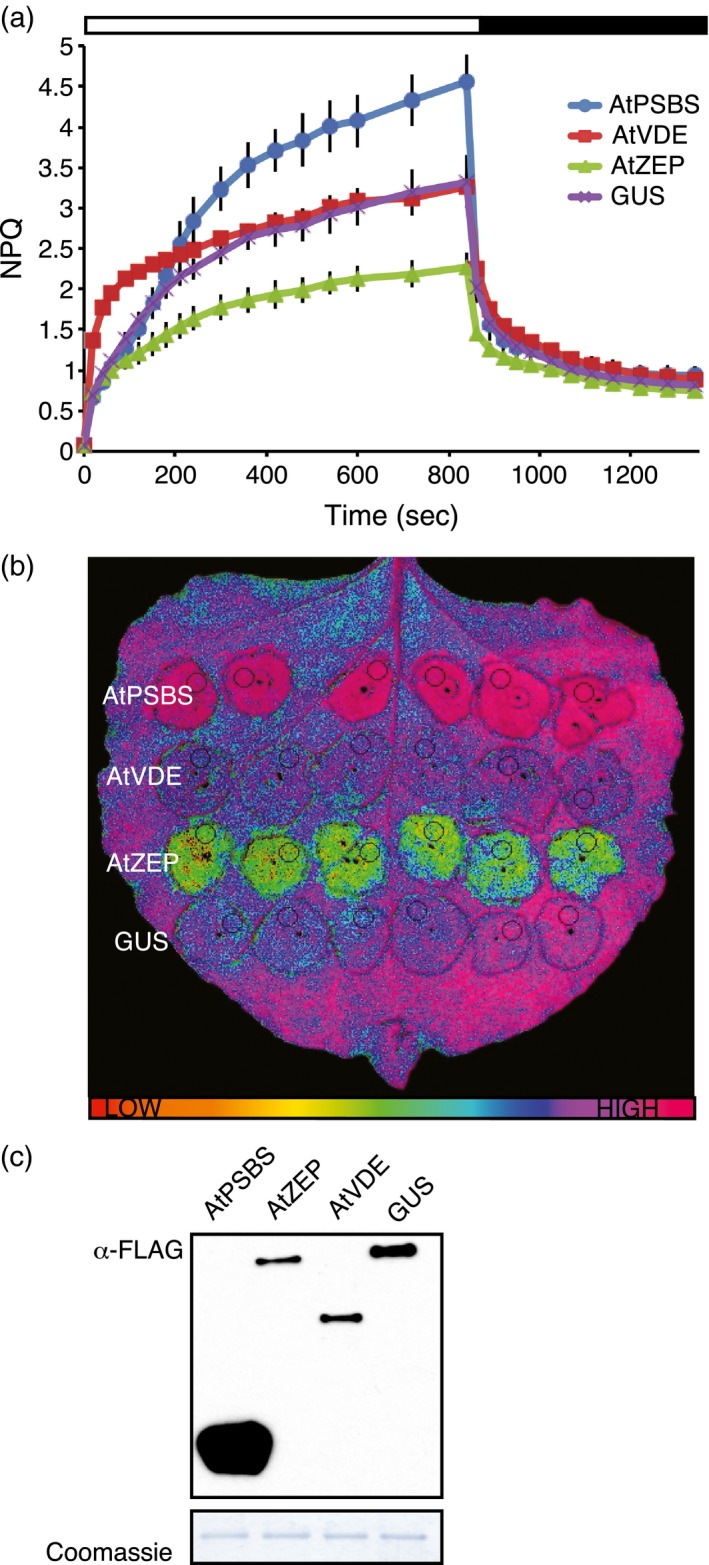
Over‐expression of *Arabidopsis thaliana* NPQ‐related genes in *Nicotiana benthamiana*. (a) NPQ measurements corresponding to leaf spots over‐expressing FLAG‐tagged *AtPSBS*,* AtVDE*, or *AtZEP* with *GUS* as a negative control. White bar indicates 600 μmol photons m^−2^ sec^−1^ actinic light exposure, whereas black bar denotes the dark recovery period. Error bars represent standard deviation (*n* = 6). (b) False‐colored image of NPQ taken with IMAGING‐PAM M‐Series (Heinz Walz) of a leaf expressing *AtPSBS*,* AtVDE*,* AtZEP*, and *GUS* 10 min after high light exposure. Rainbow bar indicates relative amount of NPQ. (c) Upper panel: Immunoblot analysis of tissue collected from panel (b) and probed with α‐FLAG. Lower panel: Coomassie loading control.

Because fully developed leaves contain mature chloroplasts and assembled photosystems, we tested whether the addition of Arabidopsis genes involved in photoprotection could alter photosynthesis in tobacco leaves at such a late stage of development. *AtPSBS*,* AtVDE*, and *AtZEP* are known to play vital roles in NPQ in Arabidopsis. We expressed these genes in fully expanded *N. benthamiana* leaves using *A. tumefaciens*‐mediated delivery and observed effects on NPQ that were consistent with results observed in stable transgenics of Arabidopsis and other higher plants (Li *et al*., 2002c; Chen and Gallie, 2012). NPQ capacity increased dramatically in leaf spots over‐expressing *AtPSBS* (Figure 1a, b), similar to the effect of increased AtPSBS protein accumulation in Arabidopsis (Li *et al*., 2002c). Leaf spots expressing *AtVDE* showed a more rapid NPQ induction than those expressing a control protein (GUS), whereas spots expressing *AtZEP* exhibited slower induction and never reached NPQ levels comparable to the GUS control (Figure 1a, b). AtZEP also appeared to increase the speed of NPQ relaxation. All expressed proteins were tagged with a C‐terminal FLAG tag (DYKDDDDK) and accumulated to detectable levels 48 h post infiltration (Figure 1c). These results suggest that by fine‐tuning the expression of these proteins, NPQ kinetics can be engineered to better match the kinetics of light fluctuation.

**Figure 2:**
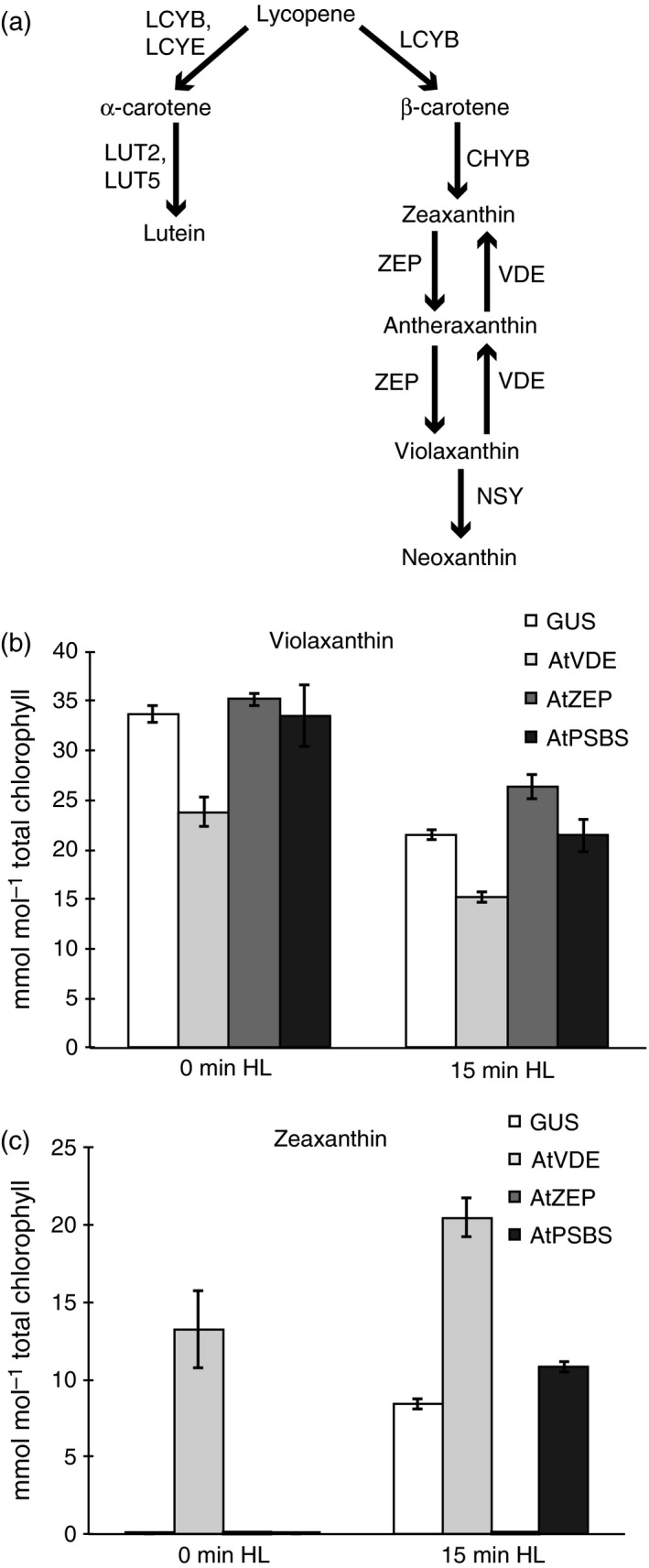
Xanthophyll accumulation in *N. benthamiana* leaf spots over‐expressing *Arabidopsis thaliana* NPQ‐related genes. (a) Schematic diagram of a portion of the carotenoid biosynthetic pathway. Lycopene β‐cyclase, LYCB; lycopene ε‐cyclase, LYCE; β‐carotene hydroxylase, CHYB; ε‐ring hydroxylase, LUT2; violaxanthin de‐epoxidase, VDE; zeaxanthin epoxidase, ZEP; neoxanthin synthase, NSY. (b, c) (b) Violaxanthin and (c) zeaxanthin accumulation in leaf spots over‐expressing *AtPSBS*,* AtVDE*, or *AtZEP* with *GUS* as a negative control at time zero and after 15 min of high light treatment (600 μmol photons m^−2^ sec^−1^). Error bars represent standard deviation (*n* = 4).

To further investigate the NPQ phenotypes described above, we analyzed pigment composition in leaf spots before and after high light treatment. AtVDE and AtZEP act antagonistically, converting violaxanthin to zeaxanthin and back, respectively, through the intermediate antheraxanthin (Figure 2a). Zeaxanthin formation is involved in both the qE and qZ components of NPQ and is crucial for effective photoprotection (Niyogi *et al*., 1998; Nilkens *et al*., 2010). Compared to the GUS control, over‐expression of *AtVDE* caused a marked decrease in violaxanthin accumulation and a concomitant increase in zeaxanthin even before high light treatment (Figure 2b, c). With zeaxanthin already present in these leaf spots, qE is poised for activation, explaining the rapid NPQ induction kinetics similar to that of Arabidopsis *npq2* plants that lack ZEP activity (Niyogi *et al*., 1998). VDE activity depends on a conformational change that occurs when the pH of the thylakoid lumen decreases in saturating light conditions (Arnoux *et al*., 2009). Thus, our finding that zeaxanthin accumulated under low light is surprising. Even after a 12‐h dark acclimation period, zeaxanthin and antheraxanthin were still detectable in leaf spots over‐expressing *AtVDE* (Figure S1). One possible explanation for VDE activity in the dark was recently suggested based on studies of *Ulva* sp. (Xie *et al*., 2013). Using various electron transport chain (ETC.) and NPQ inhibitors, Xie *et al*. (2013) showed that VDE remained active in *Ulva* sp. in low light and implicated chlororespiration‐mediated electron transport as a possible mechanism to generate sufficient lumenal acidification to activate VDE. Alternatively, an over‐abundance of AtVDE protein crowded into the tight space of the thylakoid lumen may result in higher background activity due to an increased association of AtVDE with its membrane‐bound substrate, facilitating zeaxanthin production even under low light conditions. In its inactive state, VDE is soluble in the thylakoid lumen, however, once activated it associates with the lipid MGDG and anchors to the membrane where it acts upon violaxanthin embedded in protein complexes to produce zeaxanthin (Hager and Holocher, 1994; Rockholm and Yamamoto, 1996).

Unlike VDE, ZEP is constitutively active. In leaf spots expressing *AtZEP*, zeaxanthin did not accumulate despite 15 min of high light treatment (Figure 2c). This absence of zeaxanthin explains the attenuated NPQ response to high light seen in Figure 1(a), similar to the phenotype of the Arabidopsis *npq1* mutant that lacks VDE activity and is unable to accumulate zeaxanthin in high light (Niyogi *et al*., 1998).

The de‐epoxidation state (DES) of leaf spots expressing *AtVDE*,* AtZEP*, or *GUS* also reflected the trends observed in NPQ kinetics (Table 1). At time zero, leaf spots expressing *AtVDE* had a DES of 0.380, whereas the DES of *GUS* and *AtZEP* spots was 0. After 15 min of high light treatment, the DES of all spots increased, with *AtVDE*‐expressing leaf spots still highest at 0.573, and the *GUS* control reaching a DES of only 0.319. Despite this difference in DES, both *AtVDE‐* and *GUS*‐expressing tissue achieved similar levels of NPQ, indicating that after reaching a certain threshold DES, the amplitude of NPQ is independent of DES.

**Table 1:** De‐epoxidation states of leaf spots over‐expressing various *Arabidopsis thaliana* genes involved in NPQ

	De‐epoxidation state (0.5A + Z)/(A + V + Z)	
	Time 0	Time 15 min
GUS	0	0.319 ± 0.010
VDE	0.380 ± 0.049	0.573 ± 0.023
ZEP	0	0.113 ± 0.008
PSBS	0.013 ± 0.023	0.361 ± 0.013

These results demonstrate the plasticity of the photoprotective machinery in chloroplasts even at later stages of leaf development and indicate that this transient expression method can be used to study genes involved in photosynthesis and photoprotection. Moreover, this approach can, in principle, be used to examine gene function from any photosynthetic organism for which DNA sequence information is available, including species that are genetically intractable or otherwise difficult to work with, while also providing a consistent background for direct comparisons of gene function.

### Distantly related *PSBS* genes show varying degrees of protonation‐dependent quenching *in planta*

**Figure 3:**
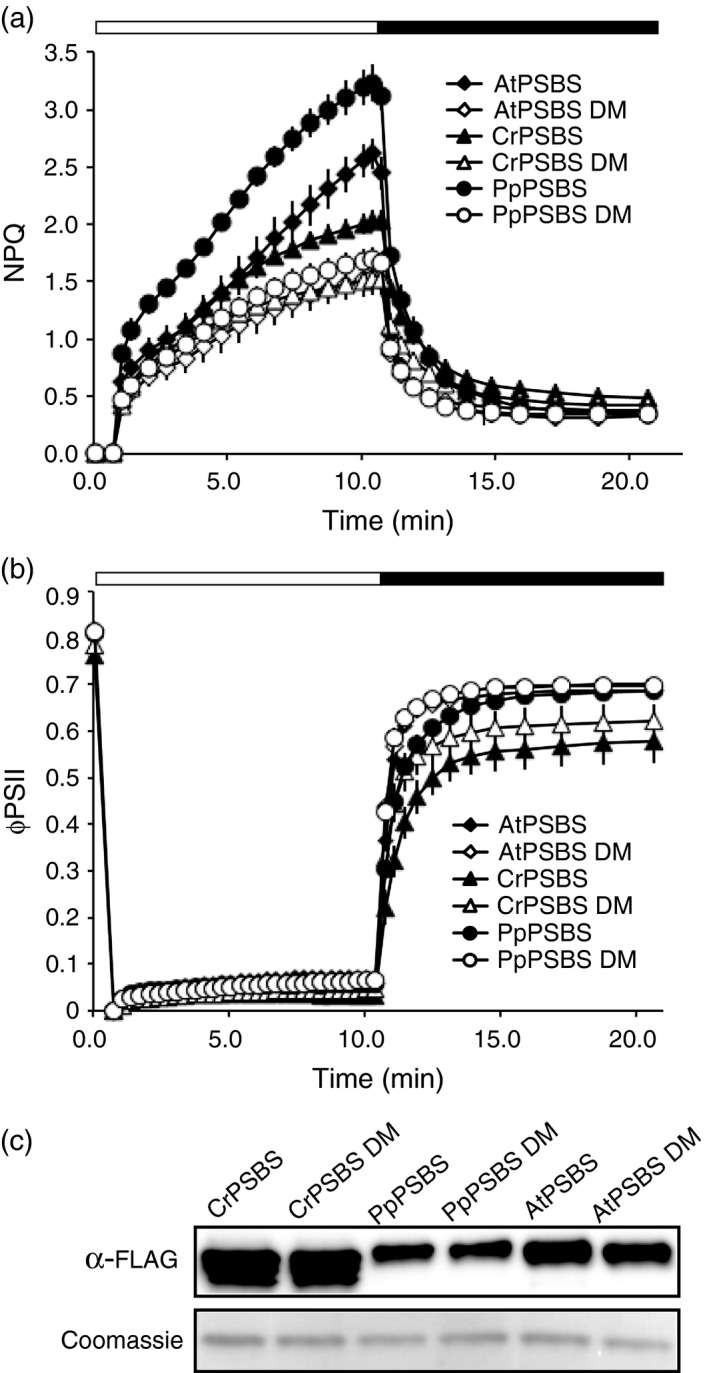
A comparison of PSBS activity from *A. thaliana*,* P. patens* and *C. reinhardtii*. (a) NPQ of *N. benthamiana* leaf spots over‐expressing FLAG‐tagged PSBS from *A. thaliana* (AtPSBS), *P. patens* (PpPSBS) or *C. reinhardtii* (CrPSBS) when exposed to 10 min PAR 600 μmol photons m^−2^ sec^−1^ (white bar) and 10 min dark recovery (black bar). DM denotes the protonation‐inactive double mutant form of each protein. Mutants are shown as open shapes. (b) ΦPSII of leaf spots containing PSBS proteins. Measurements in (a) and (b) were obtained using the Dual‐PAM‐100 (Walz) and error bars in both (a) and (b) represent standard deviation (*n* = 4). (c) Upper panel: Immunoblot probed with α‐FLAG to determine PSBS protein accumulation. Lower panel: Coomassie loading control.

As demonstrated above, *PSBS* over‐expression is an effective means of amplifying the qE component of energy dissipation. Although a lot of work has been done to characterize the role of this protein in NPQ (Brooks *et al*., 2014), there is still much we can learn about PSBS by exploring natural variation between orthologs. *PSBS* genes were cloned from *Chlamydomonas reinhardtii* (*CrPSBS*), *Physcomitrella patens* (*PpPSBS*), and *Arabidopsis thaliana* (*AtPSBS*). All PSBS proteins contain two key lumen‐exposed glutamate residues that become protonated in high light. These glutamates are crucial for PSBS activation, and when glutamine is substituted for each of these glutamates, the PSBS protein is rendered protonation‐insensitive and does not activate when plants are exposed to high light (Li *et al*., 2002b). To compare the efficacy of PSBS‐mediated NPQ amplification, *PSBS* genes from the species mentioned above were transiently expressed in *N. benthamiana*, along with protonation‐insensitive mutant versions, and were scored for their ability to induce NPQ in response to high light. All three orthologs of *PSBS* enhanced qE (Figure 3a), including CrPSBS, which has not previously been shown to function *in vivo* (Bonente *et al*., 2008). In each case, the enhanced qE was dependent on protonation of PSBS, as it required the conserved glutamate residues described in Li *et al*. (2004). PpPSBS conferred the highest levels of qE, which was somewhat surprising because *P. patens* uses both the algal‐type LHCSR and the higher plant‐type PSBS proteins to produce a robust high light response (Gerotto *et al*., 2011). In fact, the majority of quenching in *P. patens* is attributed to LHCSR1 (Gerotto *et al*., 2012), leading us to expect expression of PpPSBS alone in *N. benthamiana* to produce an intermediate level of quenching when compared to the higher plant version, AtPSBS. However, not only does PpPSBS confer higher qE than AtPSBS, it does so with a lower level of protein accumulation (Figure 3c).

Remarkably, CrPSBS also has the ability to confer quenching when expressed transiently in *N. benthamiana*, though to a lesser degree than either PpPSBS or AtPSBS. *CrPSBS* is one of the most strongly induced genes upon light exposure in *C. reinhardtii* (Duanmu *et al*., 2013; Mettler *et al*., 2014; Zones *et al*., 2015), yet previous studies found no evidence that the protein accumulates or has any functionality in its native organism (Bonente *et al*.,^2008^). Rather, *C. reinhardtii* relies on the algal‐type LHCSR protein to facilitate qE (Peers *et al*., 2009). Here we showed that when expressed in *N. benthamiana*, CrPSBS had protonation‐dependent activity that enhanced the qE response relative to the inactive mutant forms of PSBS (Figure 3a). However, both active and protonation‐insensitive forms of CrPSBS slightly reduced *F*_v_/*F*_m_ values (Table 2) and showed a lag in ΦPSII recovery after high light treatment (Figure 3b). This reduction in PSII efficiency suggests a possible disruption to membrane organization when CrPSBS accumulates in higher plants regardless of qE activation. To ensure proper localization to the plant chloroplast, we replaced the first 44 amino acids of CrPSBS with the AtPSBS transit peptide (N‐terminal 54 amino acid residues).

**Table 2:** *F*_v_/*F*_m_ values for leaf spots expressing various *PSBS* genes in *N. benthamiana. At*,* A. thaliana*;* Cr*,* C. reinhardtii*;* Pp*,* P. patens*. DM denotes a protonation‐insensitive double mutant version of PSBS

	*F*_v_/*F*_m_
AtPSBS	0.81 ± 0.01
AtPSBS DM	0.81 ± 0.00
CrPSBS	0.76 ± 0.04
CrPSBS DM	0.78 ± 0.03
PpPSBS	0.81 ± 0.01
PpPSBS DM	0.81 ± 0.01

The three versions of PSBS presented in this study all demonstrated unique NPQ kinetics and capacities. These differences can be attributed to variations in amino acid sequence rather than protein levels, as PSBS accumulation did not necessarily correspond to NPQ capacity (e.g. despite greater amounts of CrPSBS, PpPSBS was better at facilitating quenching). Examining the differences between orthologs can provide insight into how PSBS can be engineered to activate more rapidly or generate a specific amount of quenching. Based on the sequence alignment of PSBS proteins from a broad range of species, many of the differences between alleles lie in or near the lumenal loops that contain the glutamates believed to be protonated during activation (Figure S2). By exploring how changing these residues affects the pKa of activation and the intensity of quenching, we may learn more about PSBS function and ultimately discover how to tailor PSBS to produce an optimized NPQ response.

### Chromalveolate carotenoid genes modify NPQ when expressed *in planta*

In addition to manipulating expression levels of known NPQ genes and comparing the efficiency of various alleles of *PSBS*, we used the *N. benthamiana–A. tumefaciens* system to investigate the function of putative carotenoid biosynthetic genes identified by homology searches from the marine algae *Nannochloropsis oceanica* and *Thalassiosira pseudonana*. Based on bioinformatic analysis, genes homologous to plant cytochrome P450‐dependent β‐carotene hydroxylase (*NoCYP97F5*) (Nelson, 2006), violaxanthin de‐epoxidase (*NoVDE*,* TpDDE*), and zeaxanthin epoxidase (*NoZEP1*,* NoZEP2*) were cloned from *N. oceanica* and *T. pseudonana* and were expressed transiently in tobacco (Table S1). In certain cases, native transit peptides were replaced with that of AtPSBS (the first 56 amino acids) to ensure proper localization (Figure S3).

Functional annotations of genes from algae outside of the green lineage are often difficult to predict with confidence due to low sequence similarities. For instance, a BLAST search using the ZEP protein sequence from *Arabidopsis* identified two *ZEP* homologs from *N. oceanica*,* NoZEP1* and *NoZEP2*, both of which had less than 50% sequence identity to the original query (Table S1). Although both of these proteins accumulated when expressed *in planta* (Figure S4), only NoZEP1 demonstrated epoxidase activity on available substrates (Figures 5e and 6c). While this result does not eliminate the possibility that NoZEP2 has epoxidase activity, it does implicate NoZEP1 as being the enzyme involved in the interconversion of violaxanthin, antheraxanthin, and zeaxanthin (the VAZ cycle) in *N. oceanica*. This case demonstrates not only the power of this method for validating gene function, but also provides us with an opportunity to explore how these genes from other organisms can affect NPQ *in planta*.

**Figure 4:**
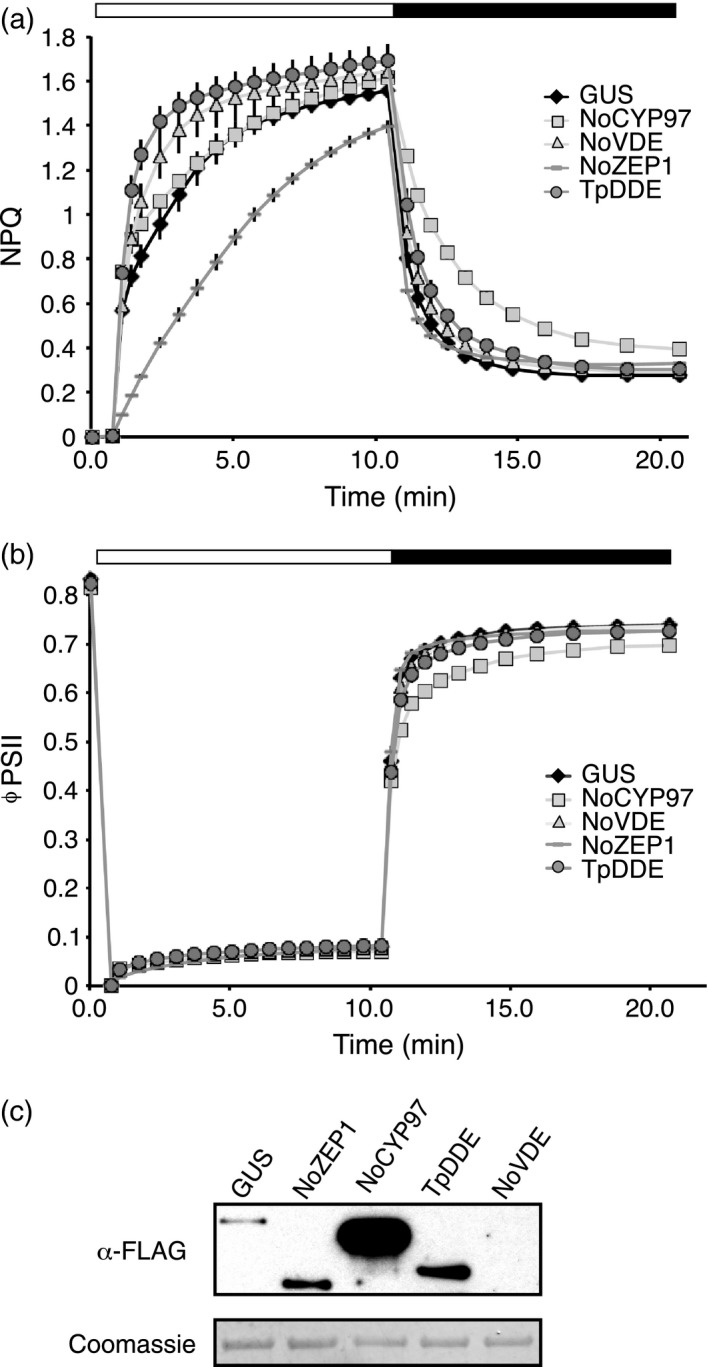
Transient expression of carotenoid biosynthetic genes from different algae in *N. benthamiana*. (a) NPQ phenotypes associated with over‐expression of genes predicted to be involved in carotenoid biosynthesis during 10 min high light exposure (600 μmol photons m^−2^ sec^−1^, white bar) and 10 min dark recovery (black bar). *NoCYP97F5* is a cytochrome P450 from *N. oceanica* that shares sequence similarity with the cytochrome P450‐type carotene hydroxylases from *A. thaliana. NoVDE* is the *N. oceanica* homolog of *AtVDE*, and *NoZEP1* is a homolog of *AtZEP. TpDDE* is an *AtVDE* homolog from *T. pseudonana*. (b) ΦPSII of leaf spots expressing genes described above under aforementioned conditions. Measurements in (a) and (b) were obtained using the Dual‐PAM‐100 (Walz). Error bars in both (a) and (b) represent standard deviation (*n* = 3). (c) Upper panel: Immunoblot probed with α‐FLAG to determine algal protein accumulation. Lower panel: Coomassie loading control.

Remarkably, when expressed transiently in *N. benthamiana*, several of these distantly related algal genes altered the kinetics of plant‐type NPQ (Figure 4a). Much like our finding that over‐expression of *AtVDE* in *N. benthamiana* caused faster induction of NPQ upon high light treatment, *NoVDE* and *TpDDE* expression showed similar trends relative to the *GUS* control, indicating an analogous function of these proteins. Similarly, over‐expression of *NoZEP1* affected NPQ by dramatically slowing its induction kinetics, though unlike *AtZEP* over‐expression, NPQ levels in leaf spots expressing *NoZEP1* eventually approached those of the GUS control after high light treatment. *NoCYP97F5* over‐expression altered the overall shape of NPQ kinetics as well as severely impeding the speed of recovery in the dark. Additionally, leaf spots expressing *NoCYP97F5* appeared to suffer an accompanying reduction in ΦPSII recovery during the dark period compared to the control (Figure 4b). All proteins were C‐terminally FLAG‐tagged and, with the exception of NoVDE, accumulated to detectable levels after 48 h of expression (Figure 4c). Although NoVDE was not detected by immunoblotting, the NPQ induction phenotype leads us to believe that some protein must have accumulated, but was perhaps below detection limits or underwent some form of C‐terminal processing.

**Figure 5:**
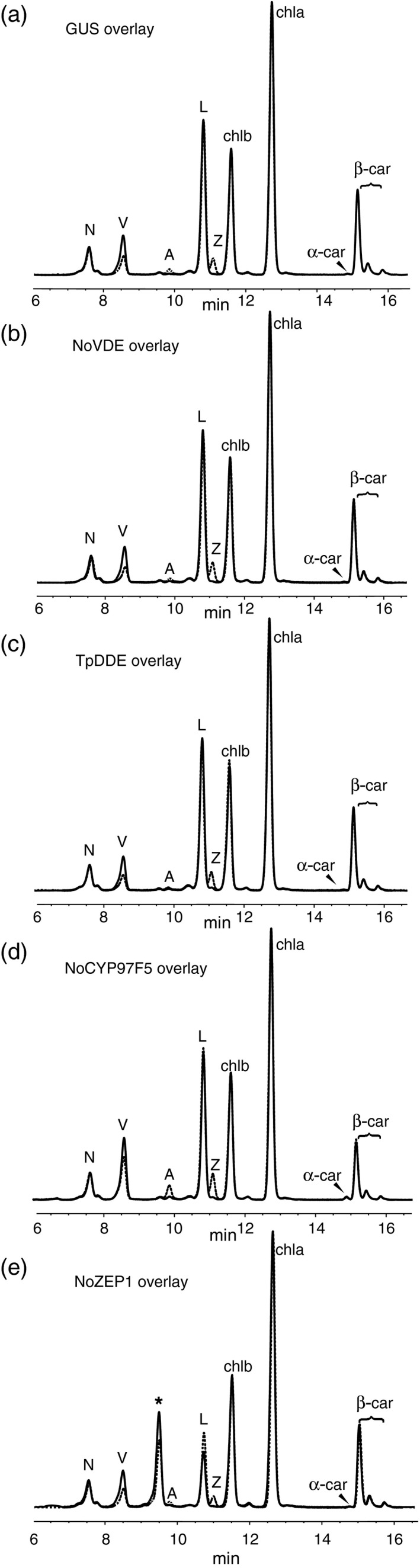
HPLC pigment profiles of leaf spots expressing predicted algal carotenoid biosynthesis genes in *N. benthamiana*. (a–e) (a) Pigments present in leaf spots expressing (a) *GUS*, (b) *NoVDE*, (c) *TpDDE*, (d) *NoCYP97F5*, and (e) *NoZEP1*. Solid black lines indicate pigment samples collected after a 20 min of dark acclimation, and dashed lines indicate pigment samples collected after 10 min high light exposure (600 μmol photons m^−2^ sec^−1^). N, neoxanthin; V, violaxanthin; A, antheraxanthin; L, lutein; Z, zeaxanthin; chlb, chlorophyll *b*; chla, chlorophyll *a*; α‐car, alpha‐carotene; β‐car, beta‐carotene. Asterisk* in (e) denotes unknown peak later identified as lutein epoxide, Lx.

**Figure 6:**
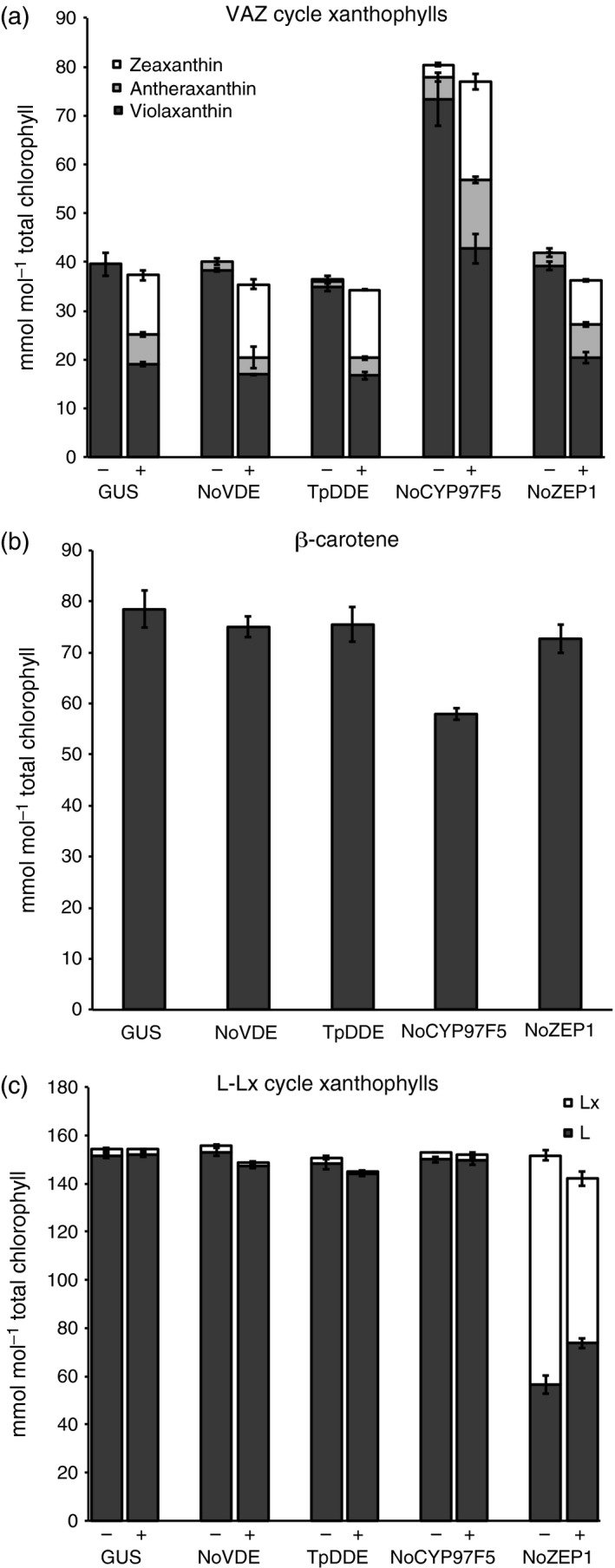
Pigment levels of *N. benthamiana* leaf tissue expressing various algal carotenoid biosynthesis genes. (a) Levels of VAZ cycle xanthophylls in leaf discs expressing algal genes listed on the *x*‐axis. ‘‐’ columns indicate samples collected before high light treatment, and ‘+’ columns indicate samples collected after 10 min 600 μmol photons m^−2^ sec^−1^. (b) β‐Carotene levels in leaf discs expressing algal genes listed on the *x*‐axis before high light treatment. (c) Levels of L‐Lx cycle xanthophylls in leaf discs expressing algal genes listed on the *x*‐axis. ‘‐’ columns indicate samples collected before high light treatment, and ‘+’ columns indicate samples collected after 10 min 600 μmol photons m^−2^ sec^−1^. L, lutein; Lx, lutein epoxide. All error bars represent standard deviation (*n* = 4).

To determine how each of the algal genes described above changes carotenoid levels, and in turn, to further understand how those changes underlie observed NPQ phenotypes, we examined pigment composition of leaf spots expressing these genes from both dark‐acclimated and high‐light‐treated samples. In the case of leaf spots accumulating NoVDE or TpDDE, pigment profiles did not vary dramatically relative to the control in either dark‐acclimated or high light treatments (Figure 5a–c). After pigment quantification, we found that NoVDE and TpDDE samples accumulated little to no zeaxanthin before high light treatment, but zeaxanthin levels were higher than that of the control after 10 min in high light (Figure 6a), as reflected by a higher DES (Table 3). These results suggest that both NoVDE and TpDDE are able to use violaxanthin as a substrate *in planta*, but they contrast with our findings that *AtVDE* over‐expression resulted in appreciable amounts of zeaxanthin even before high light treatment, and nearly double the levels of the controls after treatment (Figure 2c). This disparity suggests that NoVDE and TpDDE might be regulated differently by lumen pH than AtVDE, although both can activate rapidly in light‐saturating environments to speed the induction of NPQ (Figure 4a).

**Table 3:** De‐epoxidation states of leaf spots over‐expressing various algal genes predicted to be involved in carotenoid biosynthesis before and after 10 min high light treatment. *No*,* N. oceanica*;* Tp*,* T. pseudonana*

	De‐epoxidation state (0.5A + Z)/(A + V + Z)	
	Time 0	Time 10 min
GUS	0	0.501 ± 0.017
NoVDE	0.009 ± 0.004	0.579 ± 0.015
TpDDE	0.024 ± 0.037	0.566 ± 0.013
NoZEP1	0.014 ± 0.004	0.420 ± 0.010
NoCYP97F5	0.059 ± 0.006	0.353 ± 0.019

Transient expression of *NoCYP97F5* and *NoZEP1* led to major differences in pigment profiles when compared to the *GUS* control. Similar to studies of β‐carotene hydroxylase over‐expression in *A. thaliana* (Davison *et al*., 2002), the β‐xanthophyll pool in leaf spots accumulating NoCYP97F5 was two‐fold higher than in the control (Figure 6a). Consistent with the findings of Davison *et al*. (2002), the increase in β‐xanthophyll accumulation did not affect accumulation of α‐branch xanthophylls (Figure 6c). β‐Carotene levels, however, were 25% lower than in GUS controls (Figure 6b). This reduction of β‐carotene and the concomitant increase in violaxanthin is explained by the additional β‐carotene hydroxylase activity conferred by *NoCYP97F5* expression. It is notable that *NoCYP97F5* is the only predicted β‐carotene hydroxylase in *N. oceanica*, which lacks a homolog of the di‐iron type of β‐carotene hydroxylase (Vieler *et al*., 2012), yet it is capable of working alongside the native di‐iron and CYP97C hydroxylases in *N. benthamiana*. Violaxanthin levels were twice as high in these samples, leading to twice as much zeaxanthin production upon high light treatment. However, the increase in zeaxanthin formation did not translate into enhanced NPQ (Figure 4a) as previously observed in stable A*. thaliana* lines expressing *AtCHYB* (Johnson *et al*., 2008). In contrast to this earlier study that showed NPQ levels depend on the DES of the xanthophyll cycle pool, we found that DES was not an accurate predictor of NPQ in *N. benthamiana* leaf spots expressing *NoCYP97F5*. Despite achieving similar levels of NPQ after 10 min in high light, the DES of NoCYP97F5 samples was 0.353, whereas GUS controls had a DES of 0.501 (Table 3). The NPQ relaxation was delayed in NoCYP97F5 samples, as was the recovery of ΦPSII (Figure 4a, b). One possible explanation for the delay in NPQ relaxation is an increase in the contribution of qZ, a different zeaxanthin‐dependent component of NPQ that relaxes on a longer timescale (Nilkens *et al*., 2010) and would depend on endogenous NbZEP activity.

Most strikingly, pigment profiles of leaf spots expressing *NoZEP1* showed a marked decrease in lutein, accumulating less than half that of GUS controls (Figure 6c), and contained a major peak (denoted by an asterisk) which is absent in all other samples (Figure 5e). Because NoZEP1 was predicted to have epoxidase activity and showed a severe reduction in lutein accumulation, we hypothesized that the new peak in these samples was likely lutein epoxide (Lx). To test this hypothesis, we analyzed the pigment composition of leaf tissue from *Cucumis sativus*, a plant known to contain Lx (Esteban *et al*., 2009), and compared the retention time and absorbance spectrum of the known Lx reference against the unknown peak. Comparison of pigment profiles between *C. sativus* and *NoZEP1*‐expressing leaf spots showed that the Lx peak from cucumber clearly overlapped with the unknown peak present in NoZEP1 samples (Figure S5a). When absorbance spectra of these peaks were measured, the absorbance maxima were almost identical (Figure S5b, c), further supporting our hypothesis that the peak in question was Lx. Moreover, NoZEP1 samples showed a 20% decrease in this pigment along with a simultaneous and stoichiometric increase in lutein after 10 min in high light (Figure 6c). This inter‐conversion was likely due to the de‐epoxidase activity of endogenous NbVDE, which also converted violaxanthin to zeaxanthin (Figure 6a). This pattern of pigment accumulation is consistent with the NPQ kinetics we observed. A rapid initial rise in NPQ has been shown to depend on the presence of lutein (Pogson *et al*., 1998). Prior to high light exposure, NoZEP1 samples had 60% of their lutein sequestered as lutein epoxide (Figure 6c), simulating lutein depletion and preventing a rapid induction of qE. During high light, Lx was converted to lutein and zeaxanthin accumulated, activating qE to allow leaf spots expressing *NoZEP1* to approach NPQ levels of controls (Figure 4a).

*Nannochloropsis* lacks α‐carotenoids and consequently does not contain lutein or its derivatives (Brown, 1987; Vieler *et al*., 2012). Nevertheless, the β‐carotene hydroxylase activity of NoCYP97C has retained its substrate specificity as evidenced by the unchanged levels of lutein, derived from α‐carotene (Figure 6c). In contrast, NoZEP1 is apparently unable to distinguish between β rings of α‐ and β‐carotenoids because lutein and zeaxanthin are both epoxidized when *NoZEP1* is expressed *in planta*. This example illustrates the two‐fold benefit of screening algal carotenoid genes in this heterologous system: not only can gene function be assigned based on protein activity *in planta*, but the natural variation in substrate specificity of these enzymes can be exploited to create new pigment profiles. The resulting shifts in pigment composition can then be examined for their effect on photosynthesis and NPQ in an effort to discover new ways to optimize these pathways.

## Conclusions

In this study, we show that transient expression in *N. benthamiana* is an effective tool for manipulating dynamic photosynthesis‐related processes *in vivo*. Using this approach, we were able to manipulate NPQ kinetics in a predictable manner by over‐expression of known genes involved in NPQ induction and relaxation. Additionally, we examined the efficacy of three distantly related PSBS proteins from an alga, a bryophyte, and an angiosperm, and found that all three proteins confer additional protonation‐dependent NPQ when over‐expressed in *N. benthamiana*. While PSBS from *P. patens* showed the fastest induction and greatest increase in quenching capacity, perhaps the most striking observation was the ability of CrPSBS to contribute to qE *in planta*, suggesting that it might also have a qE‐related function in *C. reinhardtii*.

Beyond investigation of known contributors to NPQ, this method also allows for rapid identification of new components in carotenoid biosynthesis and energy dissipation from emerging model organisms. By expressing the algal genes described above, we were able to confirm predicted functions for NoVDE, TpDDE, NoCYP97F5, and NoZEP1. This study serves as proof of principle that photosynthetic genes from distantly related organisms can be assayed in this heterologous system. Furthermore, these new versions of carotenoid biosynthetic genes provide us with kinetically distinct alternatives to higher plant genes and present additional opportunities to manipulate NPQ in plants. Finally, expression of some of these algal genes in *N. benthamiana* can produce unexpected pigments that could influence photoprotection or light harvesting. For instance, *NoZEP1* over‐expression produced lutein epoxide, a pigment that has been shown *in vitro* to increase energy transfer efficiency between chlorophyll *a* molecules in a reconstituted antenna protein (Matsubara *et al*., 2007).

## Experimental procedures

### Gene sources

RNA from *A. thaliana* Columbia, *N. oceanica* CCMP1779, and *T. pseudonana* CCMP1335 was isolated using the TRIzol reagent (Life Technologies, Carlsbad, CA, USA) as per the manufacturer's instructions. cDNA was generated from these organisms with the Omniscript RT kit (Qiagen, Valencia, CA, USA), and genes of interest were amplified using the primers listed in Table S2.

### Plant growth/material

*N. benthamiana* seeds were planted in Sunshine soil mix 4 and grown in 12 h day/night cycles in a Conviron growth chamber with light intensity ranging from 60 to 80 μmol photons m^−2^ sec^−1^. Plants used in this study were between 4 and 6 weeks old.

### Plasmid construction

AttB adapter sites were added to genes of interest by polymerase chain reaction (PCR) and recombined into gateway‐compatible pDONR entry vectors using BP clonase II (Life Technologies). These pDONR vectors were used to recombine genes of interest into the pEARLEYGATE 100 destination vector using LR clonase II (Life Technologies).

### Site‐directed mutagenesis

Point mutations were introduced into PSBS using either the QuikChange Lightning Multi site‐directed mutagenesis kit (Agilent Technologies, Santa Clara, CA, USA) or the 3SM method (Follo and Isidoro, 2008). The PSBS transit peptide was added to algal genes using the splicing overlap extension PCR method (Warrens *et al*., 1997). Primers used are listed in Table S2.

### *Agrobacterium tumefaciens* transformation

*A. tumefaciens* strain GV3101 was transformed with 400 ng of each plasmid by snap freezing in liquid nitrogen for 2 min, followed by heat shock at 37°C for 5 min. Transformants were rescued in 200 μl LB with shaking at 28°C for 2–3 h and plated onto LB agar containing 30 μg/μl kanamycin and 50 μg/μl gentamycin. Single colonies appeared after 2 days of growth at 28°C.

### Transient expression in *Nicotiana benthamiana*

*A. tumefaciens* strains were resuspended in 1 ml induction medium (10 mm MgCl_2_, 10 mm MES pH5.6, 150 μm acetosyringone) and incubated for 2 h at 28°C. Cultures were then diluted to an A_600_ of 0.5 and injected into *N. benthamiana* leaves using a blunt‐end 1 ml syringe. Plants were placed under constant light (~70 μmol photons m^−2^ sec^−1^) for 48–60 h before light measurements, protein samples, and high pressure liquid chromatography (HPLC) samples were collected. In addition to technical replicates, at least three biological replicates were collected for light treatments and pigment samples.

### Chlorophyll fluorescence measurements

Video imaging was performed on detached *N. benthamiana* leaves with the IMAGING‐PAM M‐Series (Heinz Walz, Effeltrich, Germany) using 15 min of blue actinic light (600 μmol photons m^−2^ sec^−1^), followed by 10 min of dark recovery. Unless otherwise stated, samples were dark‐acclimated for 20 min prior to measurements. Photosynthetic parameters were calculated as described (Brooks and Niyogi, 2011).

The Dual‐PAM‐100 (Heinz Walz) was used to measure chlorophyll *a* fluorescence of *N. benthamiana* leaf discs expressing various constructs involved in NPQ and carotenoid biosynthesis. Unless otherwise indicated, samples were dark‐acclimated for 20 min prior to all treatments. Samples were treated with 10 min of red actinic light (660 μmol photons m^−2^ sec^−1^) followed by 10 min of dark recovery.

### Immunoblot analysis

Leaf discs (0.5 cm^2^) expressing different constructs were collected and frozen in liquid nitrogen. Samples were powdered (6.5 m/sec for 60 sec) using the cryorotor of a Fastprep‐24 (MP Bio) and resuspended in 25 μl of 3× SDS buffer. Samples were heated to 85**°**C for 3 min and then centrifuged at 14 000 rpm for 3 min to pellet cell debris. 1 μl of each sample was diluted into 6 μl of 1× SDS buffer and loaded onto a pre‐cast 10–20% Tris–glycine gel (Invitrogen) and run at 125 V for 2 h at 4**°**C. Proteins were transferred onto 0.45 μm PVDF (GE Healthcare, Pittsburg, PA, USA) using the SemiPhor semi‐dry transfer system (Hoefer Inc, Holliston, MA, USA), and membranes were blocked for 30 min at room temperature in 5% milk in TBST. Membranes were probed overnight at 4**°**C with rabbit anti‐FLAG (1:5000) primary antibody (Sigma) in TBST. Secondary antibody was donkey anti‐rabbit‐HRP (Amersham/GE Healthcare, Pittsburg, PA, USA) used at 1:25 000.

### HPLC analysis of pigments

Leaf discs (0.5 cm^2^) expressing different constructs were frozen in liquid nitrogen and powdered as described above; 100 μl acetone was added to each sample and gently vortexed for 15 sec. Cell debris was pelleted at 14 000 rpm for 3 min and the supernatant was collected. Samples were extracted a second time as described above, and supernatants were pooled to total 200 μl. Samples were analyzed with a Spherisorb 5 μm ODS1 column (Waters Corp, Milford, MA, USA) as previously described (Müller‐Moulé *et al*., 2002).

## Accession Numbers

AtPSBS: NP_175092; AtVDE: NP_172331; AtZEP: NP_851285; CrPSBS: XP_001689476; PpPSBS: XP_001778563; TpDDE: XP_002292080; NoZEP1: KU980906; NoZEP2: KU980907; NoVDE: KU980905; NoCYP97F5: KU980908.

## Supporting information


**Figure S1.** Xanthophyll levels after 12 h of dark acclimation in leaf spots transiently expressing *AtVDE* or *GUS*.

**Figure S2.** A MUSCLE alignment of mature PSBS proteins from a broad range of species.

**Figure S3.** Protein sequences of algal genes isolated and used in this study.

**Figure S4.** Immunoblot showing protein accumulation of transiently expressed *NoZEP1* and *NoZEP2* in *N. benthamiana* leaf discs.

**Figure S5.** Identification of unknown peak present in *NoZEP1*‐expressing *N. benthamiana* leaves.

**Table S1.** List of proteins and corresponding percent sequence similarity used to probe *N. oceanica* and *T. pseudonana* genomes for carotenoid biosynthetic genes.

**Table S2.** A list of primers used in this study.
