# Supplementary figures and images for: Transient expression in Nicotiana benthamiana for rapid functional analysis of genes involved in non‐photochemical quenching and carotenoid biosynthesis
Source: Plant J. 2016 Sep 15;88(3):375–86. doi: 10.1111/tpj.13268 (PMC5516181; doi:10.1111/tpj.13268)

Xanthophyll Accumulation 12h dark

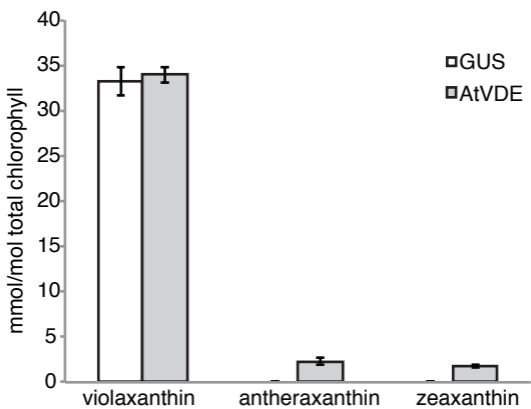

Supplement: Supplementary file 1 — Figure S1. Xanthophyll levels after 12 h of dark acclimation in leaf spots transiently expressing AtVDE or GUS. [file TPJ-88-375-s001.pdf]

$\alpha$ -FLAG

NoZEP1

NoZEP2

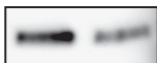

Supplement: Supplementary file 4 — Figure S4. Immunoblot showing protein accumulation of transiently expressed NoZEP1 and NoZEP2 in N. benthamiana leaf discs. [file TPJ-88-375-s004.pdf]

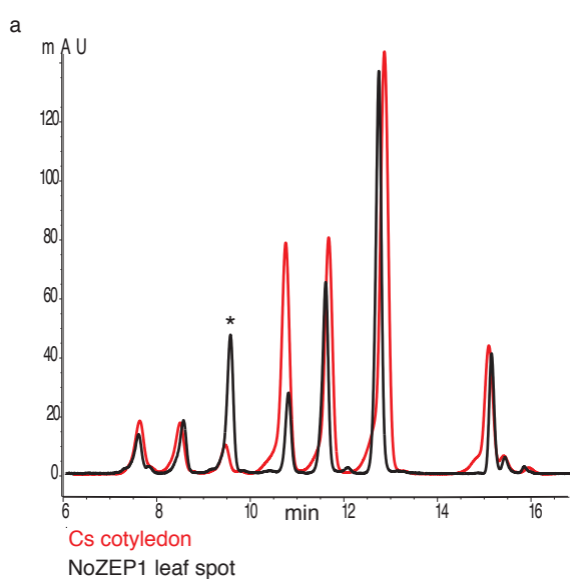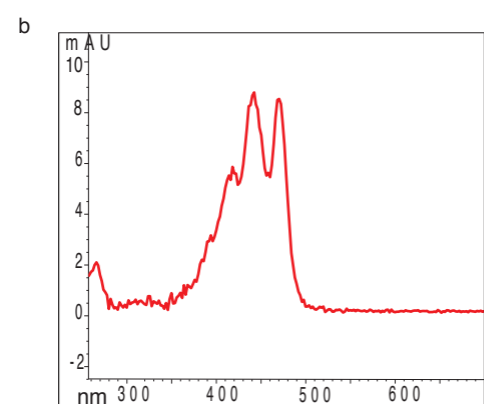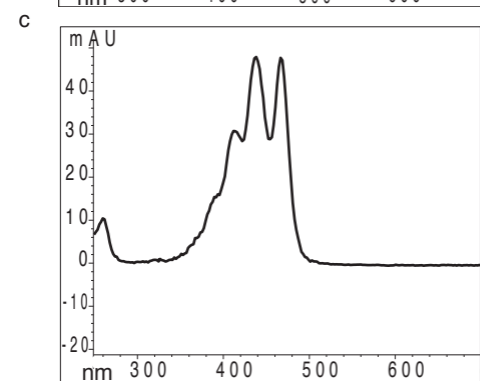

Supplement: Supplementary file 5 — Figure S5. Identification of unknown peak present in NoZEP1‐expressing N. benthamiana leaves. [file TPJ-88-375-s005.pdf]
