# Supplementary material for: Transient expression in Nicotiana benthamiana for rapid functional analysis of genes involved in non‐photochemical quenching and carotenoid biosynthesis
Source: Plant J. 2016 Sep 15;88(3):375–86. doi: 10.1111/tpj.13268 (PMC5516181; doi:10.1111/tpj.13268)
Supplement: Supplementary file 2 — Figure S2. A MUSCLE alignment of mature PSBS proteins from a broad range of species. [file TPJ-88-375-s002.pdf]

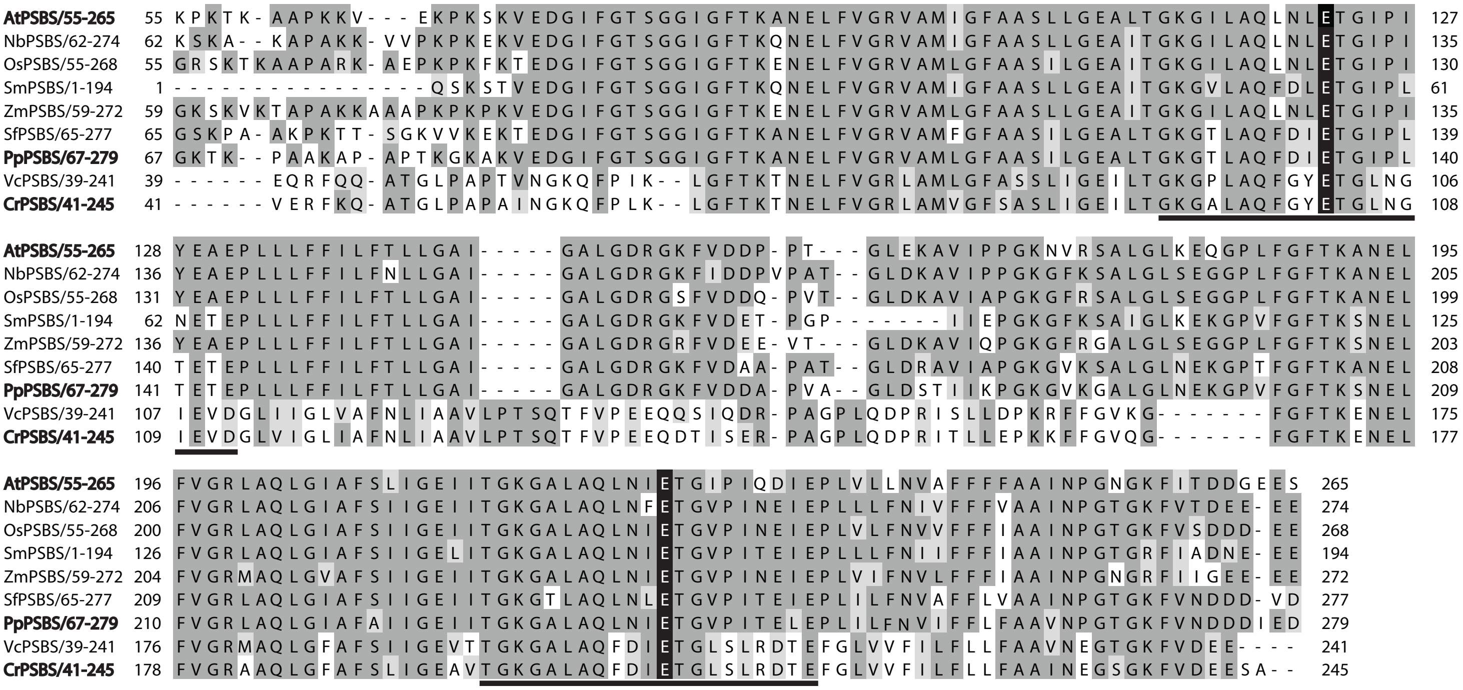
AtPSBS/55-265 55 KPKTK-AAPKKV---EKPKSKVEDGIFGTSGGIGFTKANELFVGRVAMIGFAASLLGEALTGKGILAQLNLETGIPI 127
NbPSBS/62-274 62 KSKA--KAPAKK-VVPKPKEKVEDGIFGTSGGIGFTKQNELFVGRVAMIGFAASLLGEAITGKGILAQLNLETGIPI 135
OsPSBS/55-268 55 GRSKTKAAPARK-AEPKPKFKTEDGIFGTSGGIGFTKENELFVGRVAMLGFAASILGEAITGKGILAQLNLETGIPI 130
SmPSBS/1-194 1 ----------------QSKSTVEDGIFGTSGGIGFTKQNELFVGRVAMIGFAASLLGEAITGKGVLAQFDLETGIPL 61
ZmPSBS/59-272 59 GKSKVKTAPAKKAAAPKPKPKVEDGIFGTSGGIGFTKENELFVGRVAMLGFAASLLGEAITGKGILAQLNLETGIPI 135
SfPSBS/65-277 65 GSKPA-AKPKTT-SGKVVKEKTEDGIFGTSGGIGFTKANELFVGRVAMFGFAASILGEALTGKGTLAQFDIETGIPL 139
PpPSBS/67-279 67 GKTK--PAAKAP-APTKGKAKVEDGIFGTSGGIGFTKANELFVGRVAMLGFAASILGEALTGKGTLAQFDIETGIPL 140
VcPSBS/39-241 39 ------EQRFQQ-ATGLPAPTVNGKQFPIK--LGFTKTNELFVGRLAMLGFASSLIGEILTGKGPLAQFGYETGLNG 106
CrPSBS/41-245 41 ------VERFKQ-ATGLPAPAINGKQFPLK--LGFTKTNELFVGRLAMVGFSASLIGEILTGKGALAQFGYETGLNG 108
AtPSBS/55-265 128 YEAEPLLLFFILFTLLGAI-----GALGDRGKFVDDP-PT---GLEKAVIPPGKNVRSALGLKEQGPLFGFTKANEL 195
NbPSBS/62-274 136 YEAEPLLLFFILFNLLGAI-----GALGDRGKFIDDPVPAT--GLDKAVIPPGKGFKSALGLSEGGPLFGFTKANEL 205
OsPSBS/55-268 131 YEAEPLLLFFILFTLLGAI-----GALGDRGSFVDDQ-PVT--GLDKAVIAPGKGFRSALGLSEGGPLFGFTKANEL 199
SmPSBS/1-194 62 NETEPLLLFFILFTLLGAI-----GALGDRGKFVDET-PGP-------IIEPGKGFKSAIGLKEKGPVFGFTKSNEL 125
ZmPSBS/59-272 136 YEAEPLLLFFILFTLLGAI-----GALGDRGRFVDEE-VT---GLDKAVIQPGKGFRGALGLSEGGPLFGFTKSNEL 203
SfPSBS/65-277 140 TETEPLLLFFILFTLLGAI-----GALGDRGKFVDAA-PAT--GLDRAVIAPGKGVKSALGLNEKGPTFGFTKANEL 208
PpPSBS/67-279 141 TETEPLLLFFILFTLLGAI-----GALGDRGKFVDDA-PVA--GLDSTIIKPGKGVKGALGLNEKGPVFGFTKSNEL 209
VcPSBS/39-241 107 IEVDGLIIGLVAFNLIAAVLPTSQTFVPEEQQSIQDR-PAGPLQDPRISLLDPKRFFGVKG-------FGFTKENEL 175
CrPSBS/41-245 109 IEVDGLVIGLIAFNLIAAVLPTSQTFVPEEQDTISER-PAGPLQDPRITLLEPKKFFGVQG-------FGFTKENEL 177
AtPSBS/55-265 196 FVGRLAQLGIAFSLIGEIITGKGALAQLNIETGIPIQDIEPLVLLNVAFFFFAAINPGNGKFITDDGEES 265
NbPSBS/62-274 206 FVGRLAQLGIAFSIIGEIITGKGALAQLNFETGVPINEIEPLLLFNIVFFFVAAINPGTGKFVTDEE-EE 274
OsPSBS/55-268 200 FVGRLAQLGIAFSIIGEIITGKGALAQLNIETGVPINEIEPLVLFNVVFFFIAAINPGTGKFVSDDD-EE 268
SmPSBS/1-194 126 FVGRLAQLGIAFSIIGELITGKGALAQLNIETGVPITEIEPLLLFNIIFFFIAAINPGTGRFIADNE-EE 194
ZmPSBS/59-272 204 FVGRMAQLGVAFSIIGEIITGKGALAQLNIETGVPINEIEPLVIFNVLFFFIAAINPGNGRFIIGEE-EE 272
SfPSBS/65-277 209 FVGRLAQLGIAFSIIGEIITGKGTLAQLNLETGVPITEIEPLILFNVAFFLVAAINPGTGKFVNDDD-VD 277
PpPSBS/67-279 210 FVGRLAQLGIAFAIIGEIITGKGALAQLNIETGVPITELEPLILFNVIFFLFAAVNPGTGKFVNDDDIED 279
VcPSBS/39-241 176 FVGRMAQLGFAFSIIGEVTTGKGALAQFDIETGLSLRDTEFGLVVFILFLLFAAVNEGTGKFVDEE---- 241
CrPSBS/41-245 178 FVGRAAQLGFAFSLIGEAVTGKGALAQFDIETGLSLRDTEFGLVVFILFLLFAAINEGSGKFVDEESA-- 245
