# Supplementary material for: Transient expression in Nicotiana benthamiana for rapid functional analysis of genes involved in non‐photochemical quenching and carotenoid biosynthesis
Source: Plant J. 2016 Sep 15;88(3):375–86. doi: 10.1111/tpj.13268 (PMC5516181; doi:10.1111/tpj.13268)
Supplement: Supplementary file 3 — Figure S3. Protein sequences of algal genes isolated and used in this study. [file TPJ-88-375-s003.docx]

NoZEP1

MVQLGTLLAVLFPVSSAFVFPSAPVLSSSLCSRTRSSGALSMQYGALQDKPLKVIIAGGGIGGLVLANALQRHGITYDLYEKTKEYRPFGGPIQVQSNALAALEAINKDMTQRIMDAGTTTGNRVNGLKDGMTNKWYCQFDTGAPAQKRGLPLTRVVDRPDLQNILLDYIDQSRLHTGVEVSEYSQMADGSVEVVLTNGEKVQGDVLVGADGIWSNIRGQMHKESPGKESATYSGYTCFTGVCMARPADVKEVAYKVYLAAGKYFVCSDVGKGRMQWYAMLGQPAGEKVPDGVQGAHLVKEYQGWSREVLELLEITKEEDINQRDLYDRPPLLVKGWAKGPVAVLGDAAHPMMPNLGQGGCQAVEDGYRLAQELSKITDRGDVAWALRMYEQQRLVRSSAVHGLARIASDVLFQSKLLFTNPVIGKFVGLMMTISMPLILEFLYQNVLDESDWGSARGRLSNMAAYEDQSNTYKSVREAQAALAAKETVAV-

NoZEP2+AtPSBSTP

MAQTMLLTSGVTAGHFLRNKSPLAQPKVHHLFLSGNSPVALPSRRQSFVPLALFKPMAITMATDNTLETEVLIVGVGPAGVSLAADLERRGIIDYLLVEKASSPREGGTAIGFWTNAWRCLESLGVAEGLRKSYLQGERVRIGTGQEGKELTSFSLEECDGGPHEFRYIMRSDLLRQLLKIVPKQRVAYNKAVVGFREDKGEGVVIAELSCGQKIRCKALVGADGVGSTVHKLLFPGQKLANYAGYQAIRGVATISKSGSLPPFFTFERGVVNQVWGAGVRLGTFRMTETSLYWFVTYNDQQREAGRDDDGAELLQKVRSLLAGWDARWGIDTILKNTPAQDVLRTSIGDRWPKPLGNWGQGRVTLLGDAAHPMTPNLGQGGAAGMEDALVLGELLVAALDKGDVGAETVPQALRAYEKERGRRITYLTLKSFIFGFLLQIPYSPVTFVRDNLALPLVFKPRSFLSHTKFEAPPLPSKVEKKKRRMFPLM-

NoVDE

MQKRARDQDRSSDWWKDPRATQRLLDPASAPLSAAERVKPAAAASSCASSSSSSSSSSFDRFLFSASYSMRNFKRLAFSVALPVLLSINGDSLFAGGLPHPPVAAAVDSVKVGKCLLQNCQLELARCILDPKCLANVICLNTCNNKADEVGCQIGCGDLFENEVVGQFNACALSKKQCVPRKEDDGSYPVPADSALVPSFDINSFKGKWYISAGLNPLFDIFDCQVHFFTVQGDKLYGKLNWRITEPDGEFFTRDTVQRFVQDPKFPGKLYNHDNEYLHYQDDWYILDHEPDQFIAIFYRGQNDAWTGYGGGVVYTRGPSVPPKYKERISAAFAKAGVDYAKFQDVDNTCSEQSKNPTVLRAEFARRLFLTEEQQLQEALTTARITAGNAILMEEKEAQAAVKELERAINNFERGVEEEVIKDVKVLEGEIKEVEKTFEKVIDTNLWKKNNQQNLDSLAQKLEGRPLVDPRPAPK-

TpDDE+AtPSBSTP

MAQTMLLTSGVTAGHFLRNKSPLAQPKVHHLFLSGNSPVALPSRRQSFVPLALFKPSNPVVSRTHSSVHSQQHNHVLEAHNDNMDDITFSLSARNINNEIVERIGKVTTSALLALTLSFSAITSPISGPNGDVLSSIPSANAADGAKIGLCLVKKCRVPLAKCITNPNCLANVICINSCNGKEDETGCQINCGNVFENDVVGEFNKCAVTDMTCVPQKKDDGSYPVPSKDVLVQSFDTKLWNGRWFITAGQNKLFDTFPCQVHFFTETAPGKFVGKLNWRIEEPDGEFFTRDAVQEFVQDPNNPAHLINHDNEYLHYQDDWYIVDYAADDNKEGVPPFAFVYYRGENDAWIGYGGAVVYTRDSKLPESLLPRLREAAKKVNFDFDKDFDLTDNSCKALEKGEEVVLREKFAGKMAIQTEKQLQQQAVLARTAASNTVKGEVTAVEKSLQKIEEKALAFEKELMKDVVSVEKEIVKEVEEVEKEIVQEEQKIFGGIR-

NoCYP97F5+AtPSBSTP

MAQTMLLTSGVTAGHFLRNKSPLAQPKVHHLFLSGNSPVALPSRRQSFVPLALFKPMEVFEGNPFGKAVWNLVWKLPVMKRGKPGTPIEFGDVAHVLRKNIEQIYGNEPSVDGAPLAEGEVSSMTDGTAFLGLYQYFRKYGPVYKLCFGPKSFIVLSDPTIIKHVLKTNNAAYDKGLLAEILEPIMGKGLIPADNETWKVRRRAIVPAFHQQWLEHMISLFGGCSDVLMQKLDGLAATKTDVDMENLFCSVSLDIIGKAVFNYPFDSVTKESPVIQAVYSVLKEAEHRSVTPLPYWNLPLANLVVPRLRKFKADLKVINDVLDELIEKARNSQEVTDLEDLEKRNYNKVGDASLLRFLVDLRGEDTSNKQLRDDLMTMLIAGHETTAAVLTWALFEISQKPDVRAKLQAEVDRVLGDRMPTLEDIKAMQLVRFSVAESLRMYPEPPLLIRRALEDHTLPKGTAGFEPRIIRGCDIFISIYNLHRDPDLWPKPDNFDPERYLRPHSNKAKYPDWAGFDPAQVTGLYPNEVAADFAMLPFGGGARKCVGDQFATLEATVTLAMLLRRFDFDFVGRPEDVGMFTGATIHTRNGLKMTPRRRVPAPPAATPPPATTVIDRELQPQGA-

**Figure S3.** Protein sequences of algal genes isolated and used in this study. Green highlighting indicates the AtPSBS transit peptide that was fused to several of these genes to ensure proper localization to the chloroplast.
