# Supplementary material for: Transient expression in Nicotiana benthamiana for rapid functional analysis of genes involved in non‐photochemical quenching and carotenoid biosynthesis
Source: Plant J. 2016 Sep 15;88(3):375–86. doi: 10.1111/tpj.13268 (PMC5516181; doi:10.1111/tpj.13268)
Supplement: Supplementary file 6 — Table S1. List of proteins and corresponding percent sequence similarity used to probe N. oceanica and T. pseudonana genomes for carotenoid biosynthetic genes. [file TPJ-88-375-s006.pdf]

Table S1. List of proteins and corresponding percent sequence similarity used to probe *N. oceanica* and *T. pseudonana* genomes for carotenoid biosynthetic genes. Scores and alignments were determined using BLAST.

Protein query (accession number)	Algal gene from this study (accession number)	Species	% identity	% similarity	% coverage
ZEP (NP_851285)	*NoZEP1* (KU980906)	*N. oceanica*	43	60	72
ZEP (NP_851285)	*NoZEP2* (KU980907)	*N. oceanica*	30	44	62
LUT5 (NP_564384)	*NoCYP97F5* (KU980908)	*N. oceanica*	50	67	85
VDE (NP_172331)	*NoVDE* (KU980905)	*N. oceanica*	40	56	98
VDE (NP_172331)	*TpDDE* (XP_002292080)	*T. pseudonana*	52	64	78
