# Supplementary material for: Transient expression in Nicotiana benthamiana for rapid functional analysis of genes involved in non‐photochemical quenching and carotenoid biosynthesis
Source: Plant J. 2016 Sep 15;88(3):375–86. doi: 10.1111/tpj.13268 (PMC5516181; doi:10.1111/tpj.13268)
Supplement: Supplementary file 8 [file TPJ-88-375-s008.docx]

**SUPPORTING INFORMATION LEGENDS**

**Figure S1. Xanthophyll levels after 12 hours of dark acclimation in leaf spots transiently expressing *AtVDE* or *GUS*.**

**Figure S2. A MUSCLE alignment of mature PSBS proteins from a broad range of species.**

PSBS proteins used in this study are indicated in bold. Coloring is based on Blosum62 scoring (Styczynski *et al.* 2008). Underlined letters denote putative lumenal loops, and residues highlighted in black are conserved glutamates that are protonated during high light (Li *et al.* 2002b). These glutamates were mutated to glutamines to produce the protonation-insensitive DM alleles used in this study. At (*A. thaliana*); Nb (*N. benthamiana*); Os (*Oryza sativa*); Sm (*Selaginella moellendorffii*); Zm (*Zea mays*); Sf (*Sphagnum fallax*); Pp (*P. patens*); Vc (*Volvox carteri*); Cr (*C. reinhardtii*).

**Figure S3.** Protein sequences of algal genes isolated and used in this study. Green highlighting indicates the AtPSBS transit peptide that was fused to several of these genes to ensure proper localization to the chloroplast.

**Figure S4.** Immunoblot showing protein accumulation of transiently expressed *NoZEP1* and *NoZEP2* in *N. benthamiana* leaf discs.

**Figure S5. Identification of unknown peak present in *NoZEP1*-expressing *N. benthamiana* leaves.**

**a)** HPLC pigment overlay of cucumber cotyledon tissue with *N. benthamiana* leaf disc expressing *NoZEP1*. Red line indicates cucumber sample and black line indicates *NoZEP1* sample. **b)** Absorption spectrum of cucumber lutein epoxide (Lx) peak. **c)** Absorption spectrum of unknown peak from *N. benthamiana* leaf disc expressing *NoZEP1*.

**Table S1.** **List of proteins and corresponding percent sequence similarity used to probe *N. oceanica* and *T. pseudonana* genomes for carotenoid biosynthetic genes.** Scores and alignments were determined using BLAST.

**Table S2.** **A list of primers used in this study**.
